# Unpaid carers of people with dementia and information communication technology: Use, impact and ideas for the future

**DOI:** 10.1177/14713012241249793

**Published:** 2024-04-25

**Authors:** Jacqueline Damant, Paul Freddolino, Margaret Dangoor, Bo Hu, Derek King, Raphael Wittenberg

**Affiliations:** 4905 Care Policy and Evaluation Centre of the London School of Economics and Political Science, London, UK; School of Social Work, 3078 Michigan State University, East Lansing, MI, USA; 4905 LSE, London, UK; 4905 Care Policy and Evaluation Centre of the London School of Economics and Political Science, London, UK; 4905 Care Policy and Evaluation Centre of the London School of Economics and Political Science, London, UK; 4905 Care Policy and Evaluation Centre of the London School of Economics and Political Science, London, UK; 4905 Care Policy and Evaluation Centre of the London School of Economics and Political Science, London, UK

**Keywords:** unpaid carers, people living with dementia, technology, impact, focus group

## Abstract

**Objectives:**

Several 100,000s of people living with dementia in the UK are cared for at home by a spouse or relative. Few studies have considered the ICT needs and experiences of unpaid carers. This study explores the types of ICT unpaid carers use, the ways they use ICT, the impact of ICT-use, and their ideas for how ICT could be more supportive of their role as a carer.

**Methods:**

Six focus groups with 32 unpaid carers of people living with dementia discussed their experiences of – and barriers to – using ICT. Transcripts were analysed thematically according to three types of ICT (mainstream, accessible and formal) and five functions (supporting domestic tasks, care management, monitoring, communication and information and education).

**Results:**

Participants predominantly used mainstream ICT devices such as laptops and smartphones and internet-enabled applications including videoconferencing and social media platforms to support their daily activities and assist them in their caring role. A few participants discussed using accessible devices such as memory clocks and formal telecare and care-phone services for care management and monitoring functions. Participants’ ideas for improvements centred on personalised communication applications that facilitate remote interactions and promote persons living with dementia’s independence. Others expressed concerns about the growing need to use ICT to access formal care services and the inadequacy of the ICT infrastructure in some care homes.

**Conclusions:**

Unpaid carers mostly turn to readily available mainstream ICT to support their personal and care activities. Further research is required to understand the social impact of the increasing reliance of ICT across health, social and residential care service sectors. Improved cooperation between unpaid carers, technology developers and care services providers could align ICT development to the needs and experiences of families living with dementia and assist unpaid carers with identifying ICTs that optimally support their personal circumstances.

## Introduction

Dementia is a progressive degenerative illness affecting a person’s cognitive function, causing memory impairment, behavioural and psychological changes, and for many, an eventual inability to carry out daily activities (Dementia UK, 2024; Ray & Davidson, 2014). Close to a million people are estimated to be living with dementia in the UK; approximately 82% of those living in the community are cared for by a relative or friend not formally paid for the care they provide (called hereon unpaid carers) (Alzheimer’s Research UK, 2015, 2023). Caring for someone with advanced dementia can be extremely demanding: nighttime sleep disturbances, volatile moods swings, repetitive questioning and wandering are examples of dementia symptoms that pose substantial daily challenges to unpaid carers, many of whom are themselves older adults with personal health concerns. Often as a result of their circumstances, unpaid carers experience significant deterioration in their mental and physical wellbeing (Lindeza et al., 2020).

There is a growing body of human-computer interaction (HCI) research indicating the potential for information and communication technology (ICT) to play an important role in supporting the care of people living with dementia and, in turn, alleviating some of the challenges experienced by unpaid carers. Reviews by Hassan (2020) and Caprioli et al. (2023) identified several feasibility and pilot studies of a range of digital interventions designed to support unpaid carers by facilitating their access to relevant information, emotional support, real-time care coordination, monitoring and safety systems and physical activity programmes (Austrom et al., 2015; Boessen et al., 2017; Chodosh et al., 2015; Dal Bello-Haas et al., 2014; Dam et al., 2017; Gately et al., 2020; Gaugler et al., 2016; Kales et al., 2018; Killin et al., 2018; Possin et al., 2019). Moreover, a review by Lorenz et al. (2019) identified 45 ICT devices and services available on the consumer market to support unpaid carers of people living with moderate to severe dementia: a large proportion of which support monitoring and care coordination activities. Authors also pointed to online tools available to facilitate unpaid carers’ own social, educational, and therapeutic needs.

Increasingly, HCI scholars are advocating direct input from people living with dementia and unpaid carers into the design, assessment and distribution of ICT products developed to support their needs and circumstances (Flynn et al., 2022; Hales & Fossey, 2018; Hwang et al., 2020; Rai et al., 2020; Wilson et al., 2024). Health and social care policies also emphasise the involvement of people with lived experience, including service users and unpaid carers, to advise and support empirical enquiry and the development of care services (Department of Health & Social Care, 2021; NHS England, 2024; Smith et al., 2022), including the incorporation of “*IT and assistive technology*” (Department of Health and Social Care, 2014, p. 16). Both sectors point to the potential of adopting co-production research methods for improving the suitability of – and engagement with – technologies, as well as for enhancing service users’ self-efficacy to manage their care.

Despite this objective, however, few studies of ICT-use in dementia-care contexts outline unpaid carers’ baseline technology needs, habits and preferences or demonstrate how their ICT-use guides exploration and assessment of new digital systems (Grossman et al., 2018). Knapp et al. (2015) and Sriram et al. (2021) found that unpaid carers predominantly used household ICT devices such as telephones, televisions, MP3 players, and services including home Internet and online applications for monitoring, care coordination, reminiscence, entertainment and communication functions. Brookman et al. (2023) surveyed unpaid carers’ use of GPS-enabled smartwatches, smartphones and digital picture frames for monitoring, reminder and reminiscence functions to support the care they provide. Gibson et al. (2015) noted a trend amongst unpaid carers for linking GPS and camera devices to a tablet computer or a smartphone via WiFi Internet to monitor, supervise, and support the person they cared for. Chirico et al. (2022) reported that unpaid carers relied heavily on email, social media platforms and videoconferencing applications to keep in touch with family and friends, alleviating loneliness, managing daily routines and obtaining psychological support during the COVID-19 pandemic. Given the emphasis on end-user involvement in the development of ICT services for dementia care, and the limited understanding of unpaid carers’ everyday use of ICT, this paper aims to address the gaps in knowledge by learning directly from unpaid carers of people living with dementia about their views and lived experiences of using ICT and the impact ICT-use has on their wellbeing. In addition, we explore unpaid carers’ thoughts on how they envisage ICT could be improved to facilitate the care they provide.

## Methods

This article presents findings from a series of focus groups, which form part of the wider CareTek project (LSE, 2024). The project received ethical approval from the LSE Research Ethics Committee on August 25, 2020 and was endorsed by the Association of Directors of Adult Social Services on June 24, 2021.

Recruitment to the focus groups was conducted through Adult Social Care Services at partnering local authorities and collaborating third sector organisations, who approached carers they deemed appropriate to participate. Inclusion criteria included being over 18 years of age and providing (unpaid) care for an adult currently living with dementia. We aimed to include people with varying levels of ICT literacy; carers with limited experience or knowledge of ICT were especially encouraged to take part. Written informed consent was received from all carers prior to their participation in the focus groups.

Between August 2021 and March 2022, a total of 32 participants in six focus groups across four regions of England discussed topics around their ICT-use in daily life and in support of the care they provided for a person living with dementia, the barriers to using and accessing ICT, the use of ICT during the COVID-19 pandemic and the impact ICT-use had on their quality of life and wellbeing. Each focus group concluded with a question around how participants envisaged technology could help them in any aspect of their life. Due to pandemic regulations, early focus groups were held on Zoom (Zoom Video Communications, 2023). Participants requiring care-related or digital assistance were reimbursed for additional respite services and/or individual support used to access the meetings.

Focus group participants were predominantly female (83%) and assumed to be aged over 65 years (63%). Approximately half of the participants (53%) were caring for a spouse living with dementia. Six of the participants cared for a person with dementia who resided in a care home (see Table 1). Most participants described the person they cared for as having advanced cognitive impairment and requiring high levels of support.

**Table 1.** Characteristics of focus group participants.

Focus group ID	Date mon, year	England region	Format	*N* participants	*N* female	^a^*N* 65 years+	*N* relationship to person living with dementia			*N* relatives with dementia in a care home
							Child	Spouse	Other relative	
Site 1	Aug, 2021	West Yorkshire	Virtual	7	7	5	2	4	1	0
Site 2	Sept, 2021	Greater London	Virtual	3	2	2	2	1	—	0
Site 3	Oct, 2021	Greater London	Virtual	3	2	2	1	2	—	2
Site 4	Mar, 2022	Northeast	In-person	6	5	4	0	5	1	3
Site 5	Mar, 2022	Northeast	Virtual	6	4	1	5	1	—	1
Site 6	Mar, 2022	Southeast	In- person	7	6	6	1	4	2	0
Total				32	26 (81%)	20 (63%)	11 (34%)	17 (53%)	4 (13%)	6 (19%)

^a^Several participants did not disclose their age. It was assumed participants were over 65 years when caring for a spouse and referred to being retired from formal employment.

The interviews were recorded and transcribed verbatim. Transcripts were reviewed and analysed thematically by two researchers (JD, PF), using a framework analysis approach (Gale et al., 2013). After initial familiarisation with the interview data, four transcripts were reviewed to identify the functions and the impact of ICT-use by carers, using both a deductive approach, which drew on an ICT function typology developed by Lorenz et al. (2019), and an open-coding technique to unveil new themes specific to the personal experiences of focus group participants. Researchers grouped codes into themes and agreed the resulting analytical framework which comprised five main functions: supporting instrumental activities of daily living (IADLs), care management, monitoring and safety, communication and social interaction and education and information seeking. The definition of IADLs is based on the Lawton and Brody (1969) framework, which includes communicating with others, shopping, preparing food, housekeeping and accessing transport, managing medication and finances. Subsequent analysis departed somewhat from the original parameters of the Lawton and Brody framework: communication became a standalone function and financial management was widened to include general and legal administration. The framework was then applied to all transcripts and coded excerpts were charted in a matrix in NVIVO (Lumivero, 2020).

Each ICT that participants mentioned they used, or had knowledge of, was recorded and organised into three categories, broadly based on earlier typologies of assistive technologies: mainstream ICT devices and services, accessible ICT devices and services, and formal ICT (Carretero, 2015; Gibson et al., 2015).

***Mainstream*** devices are ICTs available on the market and developed for general use with multiple applications and functionalities, such as laptop computers and smartphones. Mainstream services included the Internet and internet-enabled applications such as email and virtual assistants such as Alexa, Google and Siri (Amazon.com, 2023; Apple Inc, 2023; Google LLC, 2023).

***Accessible*** devices and services are “off the shelf” mainstream ICTs adapted for use by people with cognitive, sensory or physical limitations available for private purchase on the market. For example, participants discussed using dementia “memory” clocks and location tracking devices.

Finally, ***formal*** ICT refers to devices and services designed to assist people with cognitive, sensory or physical limitations to live independently in their own homes and are largely accessed through statutory adult health and social care services. Participants discussed using telecare services, such as pendant alarms, and telehealth services such as blood glucose monitoring applications.

## Findings

Focus groups participants were asked their thoughts on the word ‘technology’. The ensuing discussion led to participants describing the types of technologies they used, how they used them, and the impact the technologies had on them. Table 2 summarises examples of the activities and ICT devices and services participants stated they used according to three ICT-types and the five functions of the analytical framework.

**Table 2.** Summary of ICT used by unpaid carers of people living with dementia by function and type.

Function	Examples of activities	ICT type		
		Mainstream	Accessible	Formal
Supporting IADLs^a^	• Shopping	Laptop		
	• Preparing food	Smartphone		
	• Housekeeping	Email		
	• Accessing transport	Internet services		
	• Medication and financial management			
	• Legal administration			
Care management^b^	• Organise, plan personal care of person living with dementia	Smart/mobile phone	Hearing aid	Pressure (sensor) mat, “bed alarm”
	• Alleviate dementia symptoms	Tablet computer	Accessible “dementia” clock^c^	Door sensor
	• Organise formal care services	Smart speakers	“Dementia” radio	“Room alarm”
		Social media		
		Virtual assistant email		
Monitoring and safety	• Surveillance	Camera, mobile phone, mobile app	Tracking device	Pendant (wrist) alarms (telecare)
	• Personal safety	Smart watch		Smoke alarm, fire alarm
	• Health monitoring	Baby monitor		CO alarm
		Call blocker service		Blood glucose monitor (telehealth)
Communication and social interaction	• Communication	Mobile phone	Digital picture frames	Video “carephone” service^d^
	• Interaction	DVD player		
	• Maintaining connections and networks	Tablet computer		
		WhatsApp		
		Facetime		
		Zoom		
		Facebook		
Education and information seeking	• Courses	Internet		
	• Research	Laptop		
	• Information	Smartphone		
	• Employment	LinkedIn		
		Facebook		

*Notes.*

^a^IADLs: instrumental activities of daily living (Lawton & Brody, 1969).

^b^Care management refers to the actions carers undertake to organise and plan personal care and to alleviate the dementia symptoms (e.g., confusion, anxiety, sleep disturbances) and to organise formal care services (e.g., coordinating health and social care appointments, arranging transport, etc.,) for a person living with dementia (Georges et al., 2008; NHS England, 2021).

^c^Rosebud Memory Clock (Alzheimer’s & Dementia Products Ltd, 2024).

^d^(Alcove, 2024).

### Supporting IADLs

Focus group participants discussed that using mainstream ICT devices including laptop computers and smartphones, and services, such as the Internet, supported them in conducting IADLs including shopping (M1.4; F5.4; F3.5; F1.5; F4.5, F5.6, F6.1), banking and legal administration both for themselves and on behalf of the person living with dementia they care for, especially during the COVID-19 pandemic (M1.4; F2.5; M2.5; F2.2; F1.2; M1.2):

“I have a MacBook. I have an iPad. I have an iPhone…It has helped me to manage [my relative’s] affairs through on-line banking. With having power of attorney, I can control their finances very easily now, because quite often there isn’t a branch nearby… and that has been great.” (F3.5)

Carers also explained the usefulness of smartphones and tablet computers to support the general administration of their own life:

“I’ve got an iPhone in my pocket. And I have got an iPad on the side of my bed: they all speak to each other…The diary function drives my life.” (M1.5)

### Care management

Managing the care of a person living with dementia often entails helping them with everyday tasks and managing behavioural, cognitive and psychological symptoms of the disease (Georges et al., 2008; NHS England, 2021). Focus groups participants explained how care management was facilitated by ICT. For instance, one carer described how a hearing aid system linked to the television helped their relative to enjoy their leisure time and reduce their anxiety (F3.5). Participants also commented on using ICT to minimise the confusion their relatives may experience. For example, video applications (F4.5) and virtual assistant services helped reduce repetitious questioning, allowing carers some respite:

“I bought my [spouse] an Alexa for Christmas last year, because they kept asking me: ‘What time is it? What day it is?’…It has been a lifeline because now they can just say: ‘Alexa, what is the day?’ … it has taken a bit of the load off of me. (F5.6)

Some participants (F4.4) discussed employing mainstream ICT to organise activities for the person with dementia they cared for. One carer described a reminder system that they created from a virtual assistant service and a tablet computer, prompting their relative to carry out certain ADLs:

“[My] iPad has the Alexa App on there.… So, as I [recorded]: ‘[Name!] It’s tablet time.’… and: ‘It’s time for your exercises!’...It made them take their tablets and it made them move about a little bit. [It] even reminded [relative] to drink: ‘[Name]! It’s time for a drink!’” (F5.1)

Other participants described using accessible devices, designed specifically to support people living with dementia, such as clocks and radios (F4.1; F3.1) to personalise notifications of daily events and activities:

“…we introduced a Rosebud Memory Clock… [my relatives could] tell the day, the date and the time. They could [also] choose what was displayed so you could do bespoke messages on it, with graphics. For example, when the [supermarket] delivery was coming, it would have the [supermarket] van…pop up with a sound and visual display.” (F4.5)

During the focus groups, care management was also discussed in terms of coordinating care activities between family members and formal health, social and residential care services. For example, carers used social media to coordinate care amongst other family members (F2.6):

“My daughter can access the camera from her house and my partner from his workplace…I’ll get in touch with a group message, saying: ‘I’m going driving for half an hour, can you just check in on [my relative]?’ Any of them can just check in on the camera. It’s a tiny little thing that you just plug in, just sat next to the TV: so [relative] is staring right at the camera. It’s just peace of mind.” (F5.1)

One participant explained they received email updates from their relative’s care home on their mobile phone:

“I received email from the [care home] manager …I have my mobile phone with me and [if] something happens they can always reach me. I don’t worry if I am out.…Also, emails from the manager [explain] what’s going on, what we can do, what we can’t do…You can go back to the email and it’s all there…It reassures me…” (F1.3)

Some participants (M1.6) mentioned that smartphone applications helped them align with formal home care services providing personal care for their relative:

“[The care agency] told us they could put an app on my phone …I can see what time they were there, a little report of what the situation is, stuff like that. If there is a problem, or something I need to do, they just put it on the app and I get a message saying: “this – or whatever – is needed.” (M2.5)

### Monitoring and safety

Participants discussed the technological solutions they self-fashioned to ensure the safety of their relative. As one carer explained, mainstream cameras and smartphones connected to apps via the Internet were used to build surveillance systems that allowed them to monitor their relative when they were away (F5.1; M1.3; M1.6):

“We’ve got two cameras in house: one in the lounge and one in the conservatory… I downloaded an app, connected them to it and it didn’t cost me a penny…I can see what [my spouse] is doing. [My spouse] is 5 minutes away, so that gives me peace of mind...” (M1.4)

Some participants (F2.2; F1.2) mentioned their use of retail baby alarms to monitor the movements of their relatives:

“I have a camera and a baby monitor in their room…If I wanted to make sure that they were alright, I could tap into that and see them sitting in the room. At night-time I have the monitor in my bedroom so I would hear their movement.” (F4.6)

A few participants mentioned using accessible ICTs, such as a tracking device that “alerts your mobile phone” (F4.1). And a small number of carers (F3.1; F1.2) discussed employing formal telecare services to monitor the safety of their spouse or relative:

“I find it easier leaving [my spouse] … they have a [device from the council] they can press if anything happens, and somebody is on the other end of the phone…it gives me a bit of peace of mind.” (F2.1)

### Communication and social interaction

Several participants explained that they used tablet computers, smartphones, social media and communications platforms (e.g., WhatsApp, Facetime and Zoom) to keep in touch with friends and family (F6.1; F2.2; M1.3; F1.2). Also frequently arising was the theme of social support afforded by online platforms during the COVID-19 pandemic, permitting both carers and people living with dementia to interact with others when travel restrictions and social distancing edicts were in place (M1.5). A few participants conveyed that they were able to attend significant family events, such as funerals (F2.2) and weddings (F4.5), and religious services (M1.3; M1.2) via online video streaming and recording applications.

Other carers (M1.3, F1.2) described using ICT to support social interaction between themselves and their relative with dementia:

“I use Facebook and show pictures of people and photos I take on my mobile phone to my [relative]: ‘Today this’, and ‘I have got this flower in the garden…’ [When] I am with [my relative], it gives me something to talk about.” (F1.3)

One carer mentioned the benefits of employing a formal video care-phone service, provided by their council, to facilitate connections with their relative with dementia:

“My [relative] can [use the ALCOVE service] because it is all pictures, faces. They just touch the face…It’s just a regular tablet [with] just for our close-knit family” (F2.6)

### Information seeking and education

Many participants discussed using the Internet and social media platforms (e.g., Facebook) to “do research” on topics related to dementia, ICT and DIY projects (M1.4; F2.6; F2.5; F1.3; M1.3). Participants also mentioned attending online courses for a range of topics (F2.1; F3.5), including a council-run course designed for carers of people living with dementia (F2.5).

One carer explained that since their spouse entered a care home, they rely on the Internet to learn new skills:

“I got all sorts of jobs half done because I don’t know how to do them. I have started to go to the internet and I ask it questions, and it spits something up and I have a go… That has brought my self-confidence up.” (F4.4)

#### Impact of ICT use on unpaid carers

Carers discussed the positive impacts of using mainstream ICT devices and Internet services, such as online courses (F4.4; F2.5; F1.5), videoconferencing platforms (F2.5), and gaming apps (M1.6), on easing their caregiving responsibilities, addressing some of the emotional challenges of caring for a person with dementia and attending to their own physical wellbeing.

Several carers cited the chief benefit of ICT-use was achieving ‘peace of mind’ (F3.1) that they could remotely monitor - and provide a timely response to - the needs of their relatives living with dementia (F1.2; M1.4; F4.6; F2. 1; F3.1):

“We use it at as an intercom. I could speak to [them] from downstairs, I’d say: ‘[Alexa], drop in on [relative]’s bedroom.’…or ‘Do you need a cup of tea?’ [They] could just answer. [They] didn’t have to press anything. …[Also] for those times that I have to pop out [to the shops], it is very, very reassuring.” (F5.1)

Others talked about using radio, television and online games for distraction and stimulation (M1.6; F5.6) and to relieve periods of boredom and isolation that they experienced when supporting a person living with dementia (M1.3). Some carers described a sense of emotional support from using videoconferencing and social media platforms. Participants explained that taking part in online chat groups for carers of people living with dementia allowed them to “share my feelings with people” (F6.6) and to identify with others sharing common experiences (F3.5; F2.3):

“Some people would put on [the online chat]: ‘I’ve had enough. I don’t know what to do.’ Actually, I feel like that sometimes. You feel guilty about what you are thinking sometimes. You look at the chat and you think: ‘I am not the only one feeling like this. It’s normal.’ So, [the online chats] give you some comfort.” (F4.6)

Unpaid carers of people living with dementia often have physical and mental health limitations of their own (Greenwood et al., 2019); some participants discussed the value of using ICT to manage their own needs. For instance, participants mentioned that using Zoom (F2.1), smartwatches (F4.4) and formal ICT devices (e.g., pendant wrist alarms (F5.4), blood glucose monitor (M1.5)), helped them monitor their healthcare concerns. One participant described of attending online GP appointments for their own medical needs as convenient:

“…a blessing in disguise…I couldn’t take the time out because [my relative with dementia] was quite ill...it’s half an hour [to get] there, half an hour in the waiting room and this, that and the other…” (F6.1)

Others commented on the benefits of attending online exercise classes to their “own mental health” (F2.5) and physical wellbeing (F7.1). One carer discussed the positive effect of employing a baby alarm, for monitoring their relative, on the quality of their sleep and ability to “function” (F1.2). Another carer expressed that using formal telecare services for their own needs helped improve their caregiving capabilities: “…the family have talked us into getting [telecare], mainly because my balance is awful. If I fall over, which I do occasionally, my [spouse with dementia] can’t help me up anymore…” (F1.6)

Participants also mentioned the downsides of using ICT, including increased pressures and responsibilities, perceived sense of shame and exclusion, and a reduced human contact. As one participant described, using their mobile phone as a reminder service became burdensome:

“I used to have a reminder set on my mobile phone, 6 times a day, which is quite intense. Every few hours it is going off…then I would [have to] call or go and find [my relative]… It felt like it took up most of the day.” (F5.1)

Some carers commented that formal ICT services designed in part to ease some of their responsibilities, such as telecare (F1.2; F5.1; F3.6; F2.6) and care-phone systems (F4.6; M1.2), were impractical as their relatives living with advanced stages of dementia were unable or unwilling to use the devices:

“[My relative] would just randomly ring people. Then they didn’t like [ALCOVE on the tablet] looking at them, so they put it down flat. As soon as they could get rid of it, it was gone.” (F2.6)

Several carers described themselves as reluctant ICT-users (F2.3; F1.2; F2.2; F4.1; F1.1) and explained their frustration with mainstream ICT services, in terms of feeling unable to “keep up” with frequent updates (M1.4; M2.5; F3.1; F6.1; F7.1; F1.2) and of perceiving the language of some online services to be unsympathetic and overly complicated, ultimately discouraging them from utilising ICT (M1.2; F2.1; F4.1, F1.1).

Participants also voiced concerns about a perceived increase in the use of social media platforms to socialise and to access health appointments and meetings since the COVID-19 pandemic. They expressed fears that a shift to online consultations would result in a permanent reduction of in-person appointments, and in turn, a decline in appropriate service response to the care needs of their relative living with dementia (F1.2; M1.2; F1.5). One carer expressed feeling heightened pressure to be able to identify changes in care needs and to perform care tasks, which were normally addressed by formal care practitioners:

“[My relative] hasn’t seen a doctor [in over 18 months]…That puts the responsibility entirely on my shoulders to see that something needs attention and to [contact] the District Nurse and say: ‘I don’t feel comfortable doing what’s being prescribed. Is this something you can help with?’” (F2.2)

Others articulated the negative impact of meeting people virtually rather than in person, such as missing out on the camaraderie and valuable exchanges of information and advice from their peers (F1.2):

“Seeing people in person is different to seeing people on Zoom… It has happened to me where we had a really important group and I couldn’t join in because I couldn’t work [Zoom]…that is when I feel sad…” (F6.1)

#### Carers’ ideas for the future

Focus group participants were asked to reflect on the barriers to using ICT and to propose suggestions for ICT devices and services – real or imaginary – that could potentially support them in caring for their relative living with dementia. Several ideas were brought forward to assist carers to communicate with their relatives living with dementia, to improve the accessibility of ICT devices and services (M1.2; F1.3; F2.3), and to enable their continued learning of various ICT solutions and skills.

Participants discussed the challenges with communication systems, particularly in care homes during the COVID-19 pandemic, when visitation in care homes in England was severely limited. Outdated ICT networks and devices coupled with extraordinary workloads of care home staff sometimes compromised carers’ access to their relatives with dementia (F4.4; F1.3; F2.3). One carer proposed a remote digital system, which would enable them to control the devices (TV, radio, tablet computer, etc.,) of their relative living in a care home from a distance, circumventing the need to rely on care home staff and enabling a more personalised service.

“Access to… TV in my [relative’s] room in the care home… If I pressed a button here, it could appear on [their] screen there and I could talk to [them]…put pictures on, films on, etc.” (F1. 3)

Another carer envisaged a remote two-way passive communication system that could enable carers to call and check up on the wellbeing of their relatives, without the need for the person living with dementia to activate the device:

“It would have been fabulous to have [telecare] in reverse. I could dial in and speak to [them] either through a box or through the phone, without [them] having to do anything…Things like baby monitors only work within the house. [This would give] a bit more distance.” (F1.2)

One carer suggested ideas for accessible applications on virtual assistant services, which could both serve as a diversion or reminder service, promoting some independence through undertaking a meaningful activity, as well as provide carers with some respite:

“[My spouse] would like to cook but [they] lost the ability to follow the procedure…I’ve thought: ‘[They] have the Alexa; I wonder if I can get [them] used to asking Alexa what steps and ingredients you need for very simple things…’” (F5.6)

Carers discussed the poor accessibility of household devices for people living with dementia. A recurring issue was the confusion between television remotes and cordless telephones and the challenges with manipulating touchscreen devices (F1.2; M2.5, F1.5; F4.5; F3.1; F2.1). Participants discussed possible solutions such as designing simplified devices that differed in colour, shape or texture (F1.5; F4.5; F3.1). Carers highlighted a need for technology developers to consult “*carers and people who have dementia in the very basics of designing products*” (F1.5) to arrive at useable, every-day electronic devices (remote controls, televisions, kettles, toasters and washing machines) that adapt to the changing needs of people living with dementia and that could “*take a huge amount of stress and frustration off the carer*” (F3.5).

Participants concurred that there was a lack of guiding information about ICT and how it could support them. For instance, while several participants mentioned feeling overwhelmed by the choice of ICT (F3.1; F2.2), others expressed they “*don’t know what is out there*” (M1.4; M1.3). Some participants mentioned attending the focus group to learn about what technologies are available:

“I would just like to know what is a technology that could be used. Something that: ‘Oh, that’s great. I didn’t know that exists’….” (M1.3)

Overall, across the six groups, carers called for consistent, “*step-by-step*” (F1.2) information and training about ICT devices and services and how ICT could potentially help their personal situation as a carer of a person living with dementia (F2.1; M1.3).

## Discussion

In a series of focus groups, we spoke directly with unpaid carers of people living with dementia to find out about the types of ICT carers use to support them in their daily lives, how ICT-use impacts their wellbeing, and carers’ suggestions for how they would like ICT to support them. Focus group participants discussed the wide-ranging uses of mainstream ICT such as smartphone applications, virtual assistants and social media to support most care functions and personal needs. In concordance with previous research, several participants mentioned their reliance on ICT to access dementia-care information and to facilitate connections with the person they cared for, formal care agencies, and their personal social networks (Gross et al., 2021; Núñez-Naveira et al., 2016). Participants also shed light on how their ICT-use supported IADLs such as shopping and banking, which increased sharply during the pandemic, mirroring similar discussions elsewhere (Chirico et al., 2022; Gibson et al., 2019; Hicks et al., 2023; Spann et al., 2022). Some further mentioned the convenience of using ICT for organising their *personal* routines, for administering financial and legal matters and for reducing travel to face-to-face appointments. Less attention has been given in previous studies to the latter functions: a large proportion of research focuses on the effects of ICT interventions on people living with dementia, with fewer studies considering the impacts on unpaid carers (Lorenz et al., 2019; Nilsson et al., 2021).

As described elsewhere, several carers fashioned do-it-yourself solutions for monitoring and care coordination functions using internet-enabled virtual assistant services, cameras and smartphone apps (Gibson et al., 2015). In contrast, only a minority of participants described the usefulness of accessible “dementia clocks” and formal telecare and care-phone systems for monitoring and managing dementia symptoms and communication functions, despite a prime intended purpose to support dementia care. Participants’ tendency to gravitate towards mainstream ICT confirms earlier studies, which reported that the multifunctionality and practicality of smartphones, tablet computers and videoconferencing applications enabled carers to balance their personal needs with those related to the person they care for (Caprioli et al., 2023; Spann et al., 2022; Sriram et al., 2021). Also observed, was how unpaid carers turned to an array of improvised mainstream ICT solutions because they were mostly unaware of accessible and formal alternatives (Brookman et al., 2023; Gibson et al., 2019; Sriram et al., 2021).

The impacts of ICT-use on carers’ wellbeing and ability to support their relatives with dementia were mixed. Participants echoed previous findings about the benefits of social media, online carers’ groups and courses for their sense of feeling supported, informed and physically refreshed, and ultimately, for being able to continue to provide care (Boots et al., 2014; Gross et al., 2021; Leslie et al., 2020; Sriram et al., 2021). Most participants further cited gaining peace of mind from using all types of ICT to support caregiving (Caprioli et al., 2023; Chirico et al., 2022; Gibson et al., 2015; Hicks et al., 2023; Knapp et al., 2015). Also in line with (Hicks et al., 2023), some commented that such reassurance also granted them brief moments of respite and independence (Hicks et al., 2023), while others mentioned that using tracking devices and smartphones for remote monitoring functions helped assuage concerns about undertaking activities away from the home, such as paid employment (Gibson et al., 2015; Spann et al., 2022).

However, participants also described the inaccessibility, impracticality, and at times, imposition of ICT. Several older participants expressed a degree of frustration when their attempts to embrace ICT were met with complicated menus and unforgiving error messages, leaving them feeling discouraged, overwhelmed and “*left behind*” (F1.1). Participants with relatives living in care homes further conveyed their frustration when faced with the digital inadequacies of residential facilities whilst trying to support their relatives remotely during the pandemic (Chirico et al., 2022). Other participants perceived an amplification of their intensive responsibilities, when using ICT was the sole means for accessing care services. Likewise, Powell et al. (2010) previously documented carers’ fears of the increased pressures on their caregiving capacity if ICT was perceived to replace, rather than complement, existing services, resulting in a general reluctance amongst older carers to adopt ICT to support care.

Participants’ ambivalence towards ICT is in part attributable to the unique circumstances of the pandemic, which brought on additional challenges to caring for a person living with dementia (Read et al., 2024), and forced some carers to rapidly acquire a new set of digital skills (Chirico et al., 2022; Hicks et al., 2023). However, parallel findings suggest that unpaid carers commonly experience split feelings towards ICT. Amiri et al. (2023), Hwang et al. (2020) and Dai et al. (2019) recorded how carers perceived digital health services and wearable devices to be useful for communicating with care practitioners and accessing timely information and support. Similarly to current participants, the studies also reported that carers with lower digital self-efficacy felt impeded by the presentation and language of the ICT systems. Fundamentally, these seemingly paradoxical positions evoked a desire for technology to help sustain and promote carers’ contributions to the person living with dementia’s network of care (Leslie et al., 2020), pointing to a need for an improved understanding of the social and economic impacts of networked care services as carers’ reliance on digital support is likely to increase in the years to come. This could inform the development of accessible, integrated digital care systems that span across formal, volunteer and unpaid care sectors and facilitate the delivery of personalised services that enhance the caregiving capacity of families living with dementia (Chirico et al., 2022; Gibson et al., 2019; Gross et al., 2021; Wilson et al., 2024).

Participants’ ideas for how technologies could be improved mostly featured ways to personalise communication applications of existing mainstream devices, rather than envisaging new technologies specific to dementia care (Brookman et al., 2023; Leslie et al., 2020). Equally of note, were ideas for features that empowered people with dementia to engage in performing aspects of their daily activities, such as cooking or watching television, rather than applications that required carers to undertake functions on their behalf. These suggestions align with recent recommendations for developing ICT that *enable* the involvement of the person living with dementia in planning - and participating in - meaningful activities that potentially benefit both members of the care dyad (Brookman et al., 2023; Wilson et al., 2024). Participants also confirmed proposals that dementia-care ICT research would benefit from unpaid carers’ candid participation in both the development of new digital resources and the assessment of their impacts and effectiveness. Such approaches could improve the acceptability to carers of relevant ICT, sustain their use for a longer period, empower carers to develop new skills and support networks, and reduce the need for supplementary paid care services (Gross et al., 2021; Hales & Fossey, 2018; Hicks et al., 2023; Howard et al., 2020; Núñez-Naveira et al., 2016; Rai et al., 2020; Wilson et al., 2024). Furthermore, expanded investment in collaborative user-developer research and knowledge sharing programmes could improve the understanding of how ICT can support care as well as contribute to carers’ continued participation in the economy, their social lives and civic society (Spann et al., 2022), which is an important objective for society as a whole.

An important implication for policy from these results is that prolific use of ICT to deliver care services potentially contributes to widening pre-existing digital inequalities in terms of access and skill, particularly amongst older people and those with cognitive limitations. Some of participants’ difficulties with ICT-use arose from the pandemic restrictions (e.g., a reduction of in-person professional care services) and may have since been resolved as pre-pandemic activities resume. However, social isolation, device inaccessibility and the incumbering speed of technological change are characteristics of the digital divide that preceded the pandemic (Helsper & Reisdorf, 2017). Arguably, negative ICT experiences during the pandemic were intensified manifestations of the prevailing barriers to carers’ ICT-use. UK government digital inclusion policies (Department for Digital, Culture, Media & Sport, 2022, Department for Digital, Culture, Media & Sport; Department for Science, Innovation & Technology, 2017) centre on addressing the digital skills-deficit of the paid workforce, omitting the digital needs of unpaid carers and older adults who provide essential support for a growing number of people. Some participants acknowledged their ignorance of how ICT can support them and demonstrated a keen interest to develop their ICT skills, despite often listing numerous devices and services that they use daily. This highlights a gap in the availability of ICT training and familiarisation needed to help carers to access continually updated resources that are increasing available online (Powell et al., 2010). Also underlined is the urgency of care policies to better identify unpaid carers most at risk of digital exclusion and to develop mechanisms for deploying digital support for future global emergencies (Caprioli et al., 2023; Hicks et al., 2023). The focus groups discussions demonstrated that localised help for carers exists. Some participants mentioned receiving guidance, training and support from local carers’ services, volunteer organisations and personal networks which helped draw their attention to the potential usefulness of ICT for their individual circumstances. However, also evident was that the accessibility of such help was dependent on where people lived and whether they knew astute support workers who were familiar with the digital needs of unpaid carers living with dementia.

Volunteer organisations such as Carers UK (2023), Centre for Ageing Better (2024) and Age UK (2024) offer helpful information and digital skills training to unpaid carers and older people with access to the Internet. Nevertheless, our findings emphasise the need for early, and sustained, post-diagnosis dementia support (Gregg et al., 2021; Hicks et al., 2023), which includes signposting technologies that potentially provide personalised, timely assistance to families living with dementia in accordance with their changing circumstances and individual goals (Gross et al., 2021; Howard et al., 2020; Spann et al., 2022; Sriram et al., 2021). This also represents an opportunity for local governments to work jointly with the voluntary sector to provide comprehensive brokerage-type services about the range of ICT products and services available, and to offer bespoke training about how to identify, obtain and use the ICT that would optimally serve their circumstances (Gibson et al., 2019; Leslie et al., 2020).

## Limitations

The current study is limited by the small number of participants which challenges the generalisability of the findings. The focus group format may have inhibited less vocal participants, such that the views expressed may over-represent of those people who more readily shared their experiences and views. Also, in exploring the breadth of issues around ICT-use with several participants who are under significant time pressures, we were not able to explore more topics in depth.

Most importantly, persons living with dementia were not consulted in the process: their opinions and experiences were described by their unpaid carers which may reflect carers’ personal biases and perspectives rather than those of the person with dementia.

We also acknowledge the challenges presented by holding virtual focus groups for unpaid carers with less technology experience or limited access to the Internet and ICT devices. We aimed to collect the views of carers with a range of ICT experience and skill-level, with an attempt to especially recruit carers who did not use ICT, but we accept that our chosen methods likely excluded carers with the greatest technical constraints and as a result their views are not fully reflected in the findings. Finally, our strategy was affected by the timings of the initial focus groups (shortly after a national lockdown) and the need to limit potential anxieties around COVID-19.

## Conclusion

Unpaid carers of a person living with dementia mostly turn to mainstream ICT to facilitate many care activities. ICT-use can have a positive impact on carers’ emotional wellbeing and physical health, allowing them to replenish and to continue providing support. Developing tools to disseminate appropriate information and improve digital skills for unpaid carers, and adopting co-production approaches involving unpaid carers, could help sustain the vital dementia care resources unpaid carers provide.
